# The perception of injury risk and prevention among football players: A systematic review

**DOI:** 10.3389/fspor.2022.1018752

**Published:** 2022-12-07

**Authors:** Beatriz Cardoso-Marinho, Ana Barbosa, Caroline Bolling, José Pedro Marques, Pedro Figueiredo, João Brito

**Affiliations:** ^1^ Portugal Football School, Portuguese Football Federation, Oeiras, Portugal; ^2^ Research Center in Sports Sciences, Health Sciences and Human Development, CIDESD, University of Maia, Maia, Portugal; ^3^ Portuguese Institute of Sports and Youth, IPDJ, Sports Medicine Center, Porto, Portugal; ^4^ Armed Forces Hospital, Porto, Portugal; ^5^ EPIUnit - Instituto de Saúde Pública, Universidade do Porto, Porto, Portugal; ^6^ Laboratório Para a Investigação Integrativa e Translacional em Saúde Populacional (ITR), Porto, Portugal; ^7^ Amsterdam Collaboration on Health & Safety in Sports, Department of Orthopaedic Surgery and Sports Medicine, Amsterdam Movement Science, Amsterdam UMC, Amsterdam, the Netherlands; ^8^ Hospital da Luz, Lisboa, Portugal; ^9^ Physical Education Department, College of Education, United Arab Emirates University, Al Ain, Abu Dhabi, United Arab Emirates; ^10^ Research Center in Sports Sciences, Health Sciences and Human Development, CIDESD, Vila Real, Portugal

**Keywords:** beliefs, attitudes, knowledge, soccer, athletes

## Abstract

Football is associated with a certain risk of injury, leading to short- and long-term health consequences. However, the perception of football players about injury risk and prevention strategies is poorly documented. The present article reviewed the literature about perceptions, beliefs, attitudes and knowledge toward injury risk and prevention strategies in football players. An electronic search was performed in PubMed, Scopus, Web of Science, and APA PsychINFO until July 2022. Studies were eligible if they included the perceptions, beliefs, attitudes, and knowledge about injury risk and prevention in football players from any competitive level. The risk of bias was assessed in included studies using the Joanna Briggs Institute critical appraisal checklist. A total of 14 studies were included. Most football players agreed that their risk of injury is high and prevention strategies are important, however they do not intend to use some of these strategies. The most frequent perceived injury risk factors were low muscle strength, lack of physical fitness, fatigue, excessive training and type and condition of surfaces. The most frequent perceived injury prevention factors were warm-up, workload monitoring and strength and conditioning training. It is essential to acknowledge perceived injury risk factors, as well as a better understanding of how coaching and medical departments' perceptions match with players' perceptions, and a modification in the perceptions of the several stakeholders at different levels of action.

## Introduction

In football (soccer), given the popularity of the sport, the large number of players and the levels of competition, injuries are frequent (1).

Overall, the incidence of injuries in professional football players is 8.1 injuries/1,000 h of exposure for male players (2), and 6.1 injuries/1,000 h of exposure for female players (3). At the amateur level, generally, the incidence is 12.5/1,000 h of exposure (4). For both groups and competitive levels, the incidence of injuries is higher in matches than in training sessions, and lower extremity injuries have the highest incidence rates (2–4). Also, most time-loss injuries in professional football players led to an absence of up to four weeks (5).

With such numbers, football-related injuries may have a major negative impact on physical, mental and financial burden on the players and their clubs. For example, the mean cost of an injured player in a professional team has been reported for 500.000€/month (6). Also, injuries may have a significant impact on socioeconomic systems worldwide (7), thus requiring socio-ecological perspectives that consider the specific contexts and integrate comprehensive analyses at multiple levels (8).

There is a growing call to advance the translation of evidence-based sports injury prevention programs into sustained use in practice. Studies suggest that successful incidents with risk-taking may clue to a reduction in injury risk perception in sports. Conversely, an overestimation of the risk may increase the risk of injury due to the inability to make no-risk decisions (9). To implement effective and meaningful interventions toward injury risk and prevention, it is essential to acknowledge the players' perspectives on this topic.

Thus, the present systematic review aims to provide an overview of football players' perceptions, beliefs, attitudes, and knowledge regarding injury risk and prevention, based on epidemiological definitions.

## Methods

We adopted the Preferred Reporting Items for Systematic Reviews and Meta-Analysis criteria (PRISMA) to conduct this systematic review (10). The PRISMA 2020 checklist is available for further consultation in Supplementary Material S1. The protocol has been registered in PROSPERO (reference number CRD42021270395).

### Eligibility criteria

Studies were considered eligible considering the following inclusion criteria: (i) the participants were male or female football (i.e., soccer) players; (ii) the intervention included any form of football practice as competitive or amateur; and (ii) outcome measures included perceptions, beliefs, attitudes, or knowledge towards injury risk and prevention.

Articles were excluded according to the following criteria: studies not in humans or other agents involved in sports such as coaches, medical or science departments; type of studies such as editorials, comments, case reports, guidelines, reviews or conference abstracts; studies that did not apply only to football but rather other sports, including studies with other football codes; studies that did not guide or have the primary outcome of interest.

### Information sources

An electronic search was conducted in PubMed, Scopus, Web of Science and APA PsychINFO, from inception until July 2022, to identify articles assessing the perception of injury risk and injury prevention among football players.

### Search strategy

For database search, we used the following keywords: (“injury risk” OR “injury prevention”) AND (perception OR beliefs OR knowledge OR attitude) AND (football OR soccer). We did not apply limits to the search. The full search strategy is available in Supplementary Material S2.

### Selection process

According to predefined steps, two authors (BCM and AB) independently reviewed the search results and screened publications retrieved from databases. First, articles were screened by the information outlined in the title and abstract. Then, articles potentially relevant were retrieved for full-text reading and determined eligibility for the review. Disagreements between authors were solved by consensus.

### Data collection process

Two authors (BCM and AB) independently evaluated each selected article to extract information from the eligible studies. Data were compared and discussed in case of discrepancies. If necessary, the study authors were contacted to provide further explanation.

### Data items

From the eligible studies, data were extracted regarding (1) study characteristics (first author, year of publication, country, objectives, design, instrument of data collection); (2) study participants (including age, sex, level of competition); (3) team type; and (4) outcomes (perceptions, attitudes, beliefs and knowledge about injury risk and prevention strategies).

### Risk of bias assessment

To assess the risk of bias, two researchers (BCM and AB) independently applied the Joanna Briggs Institute (JBI) critical appraisal checklist according to the study design: randomized controlled trial (RCT) (11), cohort (12), cross-sectional (12), and qualitative and mixed methods' studies (13). If disagreements occurred, authors discussed them until they reached a consensus.

### Synthesis methods

We conducted a narrative synthesis of included studies. We analysed and computed the outcomes of interest concerning football players' perceptions, beliefs, knowledge and attitudes about injury risk and injury prevention for each study.

## Results

### Study selection

**Figure 1:**
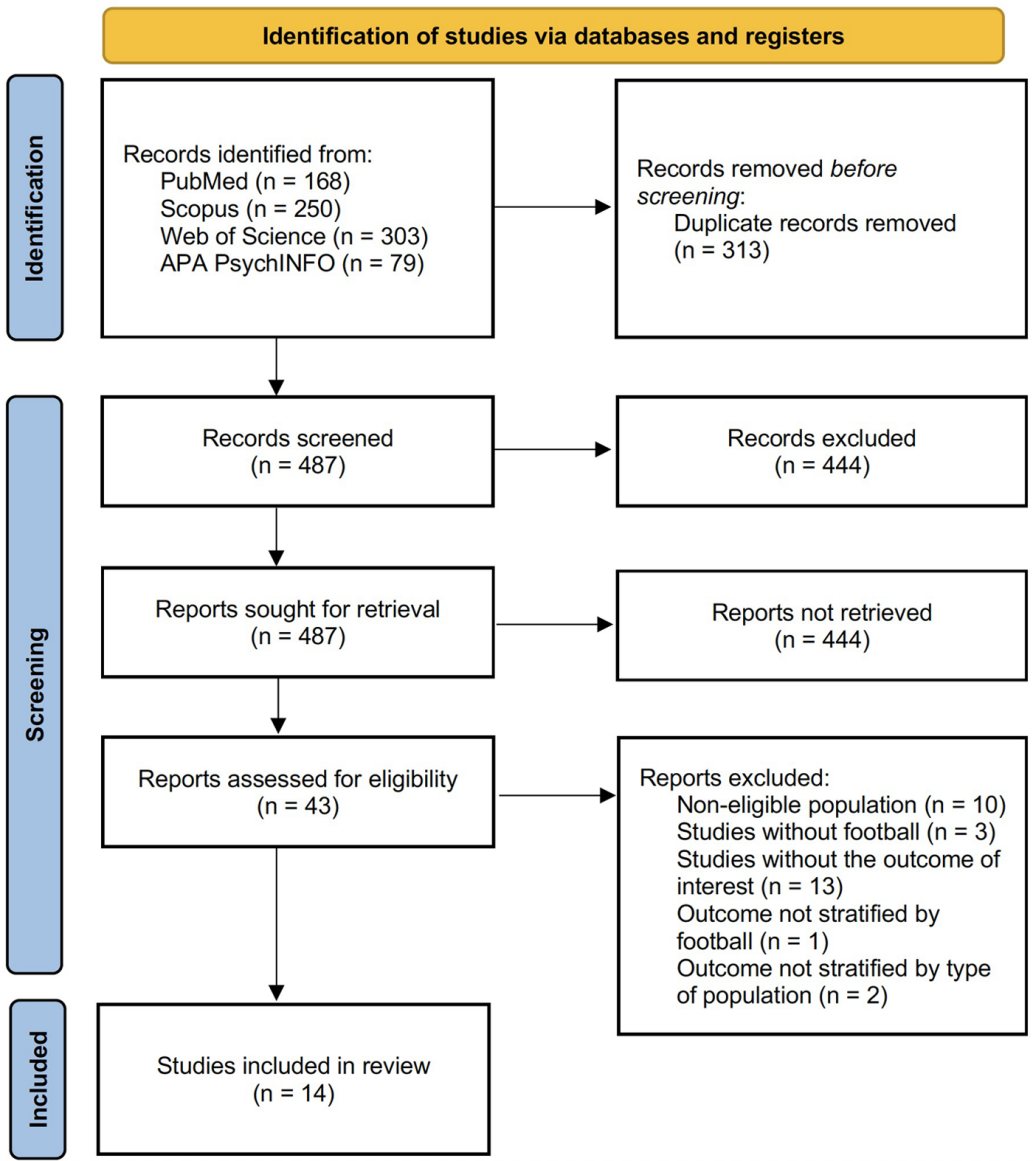
PRISMA 2020 flow diagram of included studies.

A total of 800 references were identified in the initial search from electronic databases search. After removing duplicated studies (*n* = 313), 487 studies remained. In step 1, 444 articles were excluded, and 43 studies were eligible for full-text reading, from which 29 were removed. Thus, 14 studies were included for qualitative synthesis (Figure 1).

### Study characteristics

Studies included were conducted in Qatar (14), Canada (15, 16), Norway (17), United Kingdom (18, 19), Germany (20, 21), United States of America (22), Ireland (23), Brazil (24), Brunei (25), Austria (26), and Hong Kong Special Administrative Region of the People's Republic of China (27) (Table 1).

**Table 1:** Characteristics of included studies.

Author, year	Country	Design	Participants	Age range (years)	Sex	Sample	Teams type	Objectives
Hawkins et al., 1998 (19)	United Kingdom	Cross-sectional	Professional players	18–38	M	55	5 English league clubs	To assess awareness and application of injury prevention strategies.
Kontos et al., 2004 (22)	United States of America	Cohort	Adolescent players	11–14	M/F	260 (148 M + 112 F)	18 football teams from the USA	To determine the perceived risk, risk-taking, estimation of ability, over efficacy, and previous injuries on actual injury among adolescents in sport; and to examine sex differences on these factors.
McKay et al., 2014 (16)	Canada	Cluster RCT	Adolescent players	13–18	F	258	31 football teams from the top-3 competitive levels in the Canadian league	To describe injury knowledge and beliefs and to identify the relationship between these factors, different delivery strategies of the FIFA 11 + programme and adherence.
McKay et al., 2016 (15)	Canada	Cross-sectional	Adolescent players	12–16	F	200	12 teams competing in Canadian Soccer Association	To determine the utility of the Health Action Process Approach behaviour change model in predicting intention to use the FIFA 11 + .
Zech et al., 2017 (21)	Germany	Cross-sectional	High-level players	13–35	M	139	1 high-level football club	To analyse players’ perceptions of injury prevention.
Mears et al., 2018 (18)	UK	Qualitative	Elite players	18–39	M/F	1129 (1,018 M + 111 F)	Elite football teams from 44 countries	To analyse players’ perceived links between playing surfaces and injury.
Loose et al., 2018 (20)	Germany	Cohort	Elite players	23.1 ± 4.8^a^	M	486	62 German elite teams	To reveal current opinions regarding injury prevention and return to play strategies.
Alahmad et al., 2021 (23)	Ireland	Cross-sectional	Amateur players	>18 (25.4 ± 7.7^a^)	F	158	Irish amateur winter league	To explore injury profile, opinions on risk factors and injury prevention.
Liporaci et al., 2021 (24)	Brazil	Cross-sectional	Professional players	18–37	M	100	Professional football (1st to 4th division in Brazil)	To describe the players’ perceptions towards injury risk factors and prevention strategies.
O’Brien et al., 2021 (26)	Austria	Mixed methods	Professional players	>15	M	38	Professional soccer teams (U15, U16, U18 and adult)	To evaluate the development and implementation of tailored injury prevention exercise programs.
Geertsema et al., 2021 (14)	Qatar	Cross-sectional	Elite players	>18	F	196	17 National teams	To assess knowledge, beliefs and practices of elite female footballers regarding injury prevention.
Dalen-Lorentsen et al, 2021 (17)	Norway	Cluster RCT	Elite players	IG, 17.2 ± 1.2^a^ CG, 17.4 ± 1.1^a^	M/F	85 (33 IG + 52 CG)	23 competitive youth teams in Norway	To investigate barriers and facilitators to a load management approach to prevent injuries and illnesses and their attitudes and beliefs of load management and injuries.
Som et al., 2022 (25)	Brunei	Cross-sectional	Amateur players	18–40 (23.87 ± 3.29^a^)	M/F	140 (136 M + 4 F)	Amateur football players	To assess the knowledge, attitudes, and practices on injury prevention towards lateral ankle sprain.
Weldon et al., 2022 (27)	Hong Kong Special Administrative Region of the People's Republic of China	Cross-sectional	Professional players	27.0 ± 7.0^a^	M/F	30 (17 M + 13 F)	Competitive level	To investigate the strength and conditioning practices and perspectives of soccer coaches and players.

CG, control group; F, female; IG, intervention group; M, male; NR, Not reported; RCT, randomized controlled trial.

^a^ mean ± standard deviation.

All studies were published in English between 1998 and 2022. Regarding the study design, eight studies were cross-sectional (14, 15, 19, 21, 23–25, 27), two were cluster-randomized controlled trials (16, 17), two were cohort (20, 22), one was qualitative (18), and one used mixed methods (26). The sample sizes ranged from 30 to 1,129 participants. The participants’ age ranged from 11 to 40 years old. The studies comprised elite and professional players (14, 17–20, 24, 26, 27), youth and amateur players (15, 16, 22, 23, 25), and one study involved both competitive levels (21). Five studies included both genders (17, 18, 22, 25, 27), four studies were conducted on female players (14–16, 23), and five studies involved male players (19–21, 24, 26) (Table 1).

Seven studies used a questionnaire (15, 16, 18–20, 24, 25), five studies used a survey (14, 17, 21, 23, 27), one study used a scale to analyse the perception of injury risk and injury prevention among football players (22), and one study used semi-structured interviews and focus groups (26) (Table 2).

**Table 2:** Findings of included studies.

Study	Instrument	Findings
Hawkins et al., 1998 (19)	Questionnaire	Attitudes • 51 players agreed/strongly agreed with the benefits of warming up for the risk of injury; 17 agreed with the benefits of cooling down. Knowledge • Reasons given for why cooldowns are not always performed after training or a match, were “not told to do it” and “nobody else did it”. • 51 players never wore shin pads of any type, even though 30 of these players agreed that wearing shin pads reduced the risk of a lower leg injury. • 26 players agreed that “players with poor flexibility are more likely to get injured than those with good flexibility”. • 46 players agreed that “strong muscles are important in the protection against injury”.
Kontos et al., 2004 (22)	Risk of Injury in Sport Scale	Perceptions • Lower levels of perceived risk and estimation of ability increased injury risk. • Boys reported significantly higher levels of risk-taking and lower levels of perceived risk than girls.
McKay et al., 2014 (16)	Questionnaire	Beliefs • 27.8% of female players believed that male and female soccer players had the same overall risk of injury. • 9.7%, 4.7% and 4.7% believed a warm-up would prevent muscle, knee, and ankle injuries, respectively. Attitudes • 75.8% of players considered “inadequate warm-up” a risk factor for injury.
McKay et al., 2016 (15)	Questionnaire	Beliefs • 44.0% of players believed injuries were ‘quite’ or ‘definitely’ preventable. • 50.0% believe that fracture is a very serious injury, around 40% believe concussion is very serious. • 5.5% of players believed that personal motivation/willingness to put in the effort is the most common facilitator of FIFA 11 + program, having enough time (31.5%), making the programme a routine (29.0%), and someone taking responsibility for organizing and leading the warm-up (29.0%). Players’ barriers to of FIFA 11 + programme included poor team buy-in to the programme (52.0%), finding the programme too difficult/tiring (35.5%), and low personal motivation (29.0%). Attitudes • 80.0% expected the FIFA 11 + programme to reduce injury risk but reported limited intention to use it. • 44.5% of players expected to sustain an injury during the following season.
Zech et al., 2017 (21)	Survey	Perceptions • 66.2% of respondents consider injury prevention essential or very important. Beliefs • 47.5% believed that physical contact with other players is a risk factor for lower extremity injuries, followed by physical fatigue (38.1%), environmental factors (including equipment, 25.9%).
Mears et al., 2018 (18)	Questionnaire	Perceptions • The hardness of artificial turf surfaces was perceived as contributing to almost all injuries on artificial turf. • Injuries sustained on natural turf were perceived as being caused by a wider variation of pitch conditions. Beliefs • 91.0% of players believed the type or condition of a surface could increase injury risk.
Loose et al., 2018 (20)	Questionnaire	Beliefs • 87.3% believe in injuries as a severe problem. Attitudes • 82.5% revealed a high interest in injury prevention. Knowledge • Little regeneration, a low level of fitness, and previous injury are the most frequently cited causes for injuries in elite football.
Alahmad et al., 2021 (23)	Survey	Perceptions • 53.4% not perceived increased joint mobility as a risk factor for injury. Beliefs • An inadequately treated previous injury in the same body part (56.8%) and fatigue (43.6%) were the strongest factors for increasing the risk of injury. • 7.9% believed that specific playing positions increase the risk of injury. • 74.1% did not believe that menstruation could affect the risk of injury. Attitudes • The leading prevention strategies were knowledge about the cause of injury (28.1%), and adequate warm-up (54.8%).
Liporaci et al., 2021 (24)	Questionnaire	Perceptions • The leading risk factors included poor muscle strength/power; poor rest/sleep; a short interval between matches; a high number of matches in season; and excessive training. • More than ¾ of football players considered the following strategies as being effective in reducing injury risk: workload monitoring; warm-up; lumbopelvic stability training; proprioceptive training; functional training; monitoring diet; flexibility training; and conventional strength training.
O’Brien et al., 2021(26)	Semi-structured interviews and focus groups	Knowledge • The key implementation barriers of an injury prevention programme were scheduling changes (60.0%), along with managing players workload (40.0%). • The key implementation facilitators of an injury prevention programme included programme adaptability (86.0%) and club facilities (86.0%) (infrastructure and equipment).
Geertsema et al., 2021 (14)	Survey	Perceptions • 53.0% of players understand low muscle strength as the most common cause of injuries; 49% poor pitch quality; 48% artificial turf. • 91.0% perceived impact of player motivation and 87.0% the coach's attitude as barriers and facilitators on injury prevention programs. Beliefs and attitudes • Despite players knowing that the risk of injury was high, their beliefs regarding the importance of injury prevention and actual practices relating to specific injury regions were low. Knowledge • 80.0% of players in this group of elite female footballers indicated that their risk of injury is moderate or high.
Dalen-Lorentsen et al, 2021 (17)	Survey	Beliefs • 90.0% strongly believed that load management could help reduce injury risk. Attitudes • 48.0% considered footballers to be at high risk of injuries in general, and 55.0% considered footballers to be at increased risk of overuse injuries. • Only 10.0% of players thought footballers to be at increased risk of illnesses. • 28.0% of players were willing to spend more than 10 min per week on a load management intervention, even if they thought the intervention could reduce injury. If a load management intervention could increase players’ performance, more than two-thirds (70.0%) were willing to spend more than 10 min per week doing it.
Som et al., 2022 (25)	Questionnaire	Perceptions • 84.2% of the participants perceive that injury prevention is very important. Beliefs • Participants’ beliefs on the causes of their lateral ankle sprain were lack of physical fitness (84.2%) and contact with other players (75.7%). Attitudes • Stretching (81.4%), specific warm-up training (79.3%) led the injury prevention practices that amateur players actively use to prevent lateral ankle sprain.
Weldon et al., 2022 (27)	Survey	Perceptions • 47.0% of the players perceived strength training as one of the biggest factors for reducing injuries. Beliefs • 23.0% of the players desired education on strength and conditioning training as one of the improvements for current provisions. Attitudes • 33.0% of the players reported core and 27% squat and variations exercise for injury prevention. Knowledge • 67.0% of the players reported that the strength and conditioning training is very important for reducing injuries.

### Risk of bias in studies

The included studies presented several methodological limitations. In RCT studies (11), the most common issues identified were the lack of information on randomization, allocation concealment and blinding. In cohort studies (12), most of the concerns were related to the report of factors and strategies to deal with confounding, the follow-up of participants and statistical analysis. In cross-sectional studies (12), the majority of issues identified were linked to the report of factors and strategies to deal with confounding, and the validity of the instruments used to measure the outcomes. In qualitative (13) and mixed-methods studies (13), studies lacked the statement of the influence of the researcher on the research. The assessment of the risk of bias in the studies is available in Supplementary Material S3. There was an agreement between authors when assessing the risk of bias.

### Results of individual studies

The detailed findings regarding the perceptions, beliefs, attitudes and knowledge of injury risk and prevention among football players are described in Table 2.

#### Gender differences

Deeming to the perceptions between genders, McKay et al. (16) reported that 27.8% of female players believe that male and female soccer players have the same overall risk of injury. On the other hand, Kontos et al. (22) reported that boys (11–14 years) described significantly higher levels of risk-taking and lower levels of perceived risk than girls (Table 2).

#### Injury risk factors

Concerning the perceptions about injury risk, most football players agreed that their risk of sustaining an injury was moderate to high and players expected to sustain at least one injury during the following season (14, 15, 17). Most football players believed injuries are a severe problem (20). Regarding overuse injuries, half of the players considered to be at high risk, and 10% of the players thought football players have an increased risk of illnesses (17). Considering injury severity, 50% and 40% of the players believed fractures and concussions to be very serious injuries, respectively (15). Also, Kontos et al. (22) noted that lower levels of perceived risk increased injury risk.

##### Intrinsic factors

The most frequently cited intrinsic injury risk factors were poor muscle strength (14, 24), lack of physical fitness (20, 25) and fatigue (21, 23) (Table 3). In the study of Alahmad et al. (23), joint mobility and menstrual regulation were not reasons to increase the risk of injuries in female athletes.

**Table 3:** Summary of injury risk and prevention factors.

**Injury risk factors**	
Intrinsic factors	
Lower levels of perceived injury risk	(22)
Lack of flexibility	(19)
Low muscle strength	(14, 24)
Lack of physical fitness	(20, 25)
Lack of rest and sleep	(24)
Fatigue	(21, 23)
Previous injury	(20)
Inadequate treatment of a previous injury	(23)
Extrinsic factors	
Training	
Inadequate warm-up	(14)
Excessive training	(20, 24)
Short interval between matches	(24)
High number of matches	(24)
Specific training positions	(23)
Wearing shin pads	(19)
Contact with other players	(25)
Surface	
Type and condition of a surface	(14, 18)
Artificial turf	(14, 18)
**Injury prevention factors**	
Intrinsic factors	
Motivation	(14)
Diet	(24)
Knowledge about the injury cause	(23)
Extrinsic factors	
Training	
Warm-up	(16, 19, 23–25)
Cooling down	(19)
Stretching	(25)
Physical contact	(21, 25)
Workload monitoring	(17, 24, 26)
Strength and conditioning training	(19, 24, 25, 27)
Lumbopelvic stability training	(24)
Proprioceptive training	(24)
Functional training	(24, 25)
Flexibility training	(19, 24)
Other	
Adequate equipment	(21)
Specific injury prevention programme (e.g., FIFA 11+)	(15)
Coach's attitude	(14)

##### Extrinsic factors

The most commonly mentioned extrinsic injury risk factors for injury risk were excessive training (24), the type or condition of a playing surface and specifically the artificial surface (14, 18) (Table 3). Concerning the equipment, the study of Hawkins (19) found that in training, 51 players (out of 55) never wore shin pads, even though 30 of these players agreed that wearing shin pads reduced the risk of a lower leg injury.

#### Injury prevention factors

Despite players knowing that the risk of injury was high, their beliefs surrounding the perception of injury prevention and actual practices were low (14, 15). Players seem to be interested in injury prevention strategies and consider them very important (20, 21).

However, for example, a study of McKay et al. (15) showed that, besides the expectation to reduce the injury risk with the FIFA 11 + program, players reported a limited intention to use it. Having enough time, making the programme a routine, and having someone taking responsibility for leading the prevention programme were facilitators perceived as necessary for players (15). Moreover, 60% of the players reported poor team support of the program, finding the programme too complex, and scheduling changes by club officials, soccer federations and team staff as key barriers to the injury prevention program's implementation (15, 19, 26) (Table 2).

##### Intrinsic factors

Motivation (14), diet control (24) and knowledge about the cause of injury (23) were the intrinsic aspects identified to injury prevention (Table 3).

##### Extrinsic factors

The most frequent players' features for preventing injuries were a warm-up (16, 19, 23–25), strength and conditioning training (19, 24, 25, 27), and workload monitoring (17, 24, 26) (Table 3).

## Discussion

In the present systematic review, we summarized the evidence regarding football players' perceptions, beliefs, attitudes and knowledge toward injury risk and prevention. The injury risk is multifactorial: it entangles a match between the intrinsic and extrinsic factors. On this road, we should be aware of biomechanical, anatomical, hormonal, physiological, psychological, social, and neuromuscular factors that involve and evolves the athlete (28). Therefore, it is urgent to reflect that intrinsic and extrinsic risk factors should be studied and monitored by the technical and medical teams.

Verhagen et al. (29), explain the leadership and communication to enhance health and performance in elite sports: a multidisciplinary team is required to follow the road around all of these factors that are perceived by the athlete as the risk of injury.

Lack of muscle regeneration, densely packed games in a season, inadequate workload management, and inadequate warm-up were commonly cited extrinsic risk factors for injury (16, 17, 19, 21, 23, 24). Players perceived artificial turf or uneven terrains as a possible extrinsic factor for injury, and players considered wearing shinpads to reduce the risk of injury (15, 19, 20, 22). Since the extrinsic factors were important perceived risk factors for injury, they can be monitored during the season. These factors could be used in athlete screening to target preventive interventions (30).

Overall, one of the most frequently cited intrinsic injury risk factors was poor muscle strength (14, 24), despite the already known evidence of the effectiveness of neuromuscular training strategies in reducing injury in football players (31). For example, one study conducted in female football players showed that a 15-minute neuromuscular exercise programme reduced the rate of ACL injuries, severe knee injuries and overall injuries (32).

In this systematic review, female players reported significantly lower levels of risk-taking and higher levels of perceived risk than male players. Nevertheless, gender-related risk factors show female populations to have a higher predisposition to ACL injury than males (33). However, there are no evidence-based guidelines around hormonal regulation and the injury risk for female athletes for practitioners to apply (34, 35). Particularly, in the area of injury risk, further studies are needed.

However, the lack of uptake and current maintenance of such programs is an ongoing concern. For instance, high compliance with the 11+, an injury prevention programme developed by FIFA targeting to reduce the sway of intrinsic injury risk factors in football, led to decreases in injury rates and time loss in football players (36). Also, players with high compliance with neuromuscular training programs significantly reduced ACL injury rates compared with players with low compliance (37). This highlights the importance of consistency and compliance with injury prevention training.

In this study, although most studies reveal that football players have the perception of their higher injury risk, their motivation and attitude to pursue prevention programs were limited. Lack of personal, coach and team motivation were cited as the main reasons for that (15).

Currently, significant deterioration in team and player compliance may occur throughout the season (31, 38). If injury awareness was given a similar weighting in elite sports as in any other highly physical occupation, the potential benefits and long-term health improvement could be significant (39). Therefore, the focus on implementation is critical to influencing knowledge, behaviour change, and sustainability of evidence-informed injury prevention practice. In future dissemination of injury prevention programs, players' reluctance to sustain exercise protocols should be addressed as a potential barrier to implementation (40).

Van der Horst et al. (41) studied the key issues in motivating football players to adhere to the Nordic hamstring training programme to decrease the risk of hamstring injuries. The issues were knowledge of the programme and personal motivation. Coaches and medical departments also cited personal enthusiasm and consensus with team staff to encourage adherence to the programme (41).

Moreover, the main enablers for players to implement a load management approach were scientific evidence for improved performance (88%) and mitigation of injuries and illnesses (84%), and a positive attitude of the coach towards it (86%) (17). This aligns with Andersson et al*.* (37), which established a link between player motivation and coach motivation.

Though injury prevention programs might be effective, there is a need to ensure the real vision of all stakeholders for failing to adhere to them. Players reported their motivation and the cooperation of the coach as facilitators (14, 15).

Therefore, if players have a high interest in injury prevention (22, 23, 25, 27) and firm beliefs about the warm-up (18, 21, 36, 38), workload management (19, 38), flexibility training (19, 24) and strength training (19, 24, 27) as well as diet (38), a multidisciplinary team can address the need of the injury prevention programs.

However, there is still a lack of knowledge regarding the quantity and quality of coach-led injury prevention plans and the relative impact on players' performance. Therefore, injury prevention efforts need to be built around athletes' behaviours to be effective. Consequently, there is a need to know and understand more about the behavioural aspects related to injury occurrence.

Also, it is vital to identify reasons and perceptions for increasing adherence to injury prevention programs in real-world settings by including the limitations and barriers throw players' vision. It is now understood that sports injury interventions will not have a significant public health impact if they are not widely accepted and adopted by all stakeholders (42). Moreover, real-world implementation of injury prevention interventions and evaluation of their effectiveness needs to consider the wide-ranging environmental context in which they are introduced.

The most frequently quoted injury risk issue was the lack of muscle regeneration with a short break between matches and a high number of matches in a season (22, 38). Though, players strongly believed that workload management could support reducing injury risk.

Alongside the lack of medical support (22, 36), it's important to reflect on the periods of over-scheduling during the season, in which the player's training load increases, cutting player recovery time. Furthermore, all stakeholders should help in the schedule and consider the moments of injury risk reduction, as well as awareness about the management of training conditions, such as artificial turf and field conditions, since they were perceived as a common cause of injuries (16, 20).

Concerning the agenda, players described scheduling changes as a significant hurdle for the injury prevention session (15, 17, 19, 26). Although at the same time, the agenda is problematic for the strength and conditioning training programs, the planning and integration of soccer practices that could promote injury prevention, such as small-sided games, is a worthwhile opportunity (43).

### Implications for practice

Sports injuries can result in significant setbacks, pain, social isolation, depression, disability and loss of income being some of them (44). It can also predispose athletes to degenerative disorders, such as osteoarthritis (44). A preventive approach is paramount, and exercise can be an effective tool to prevent sports-related injuries. Thus, the factors hindering athletes' injury awareness from achieving occupational health standards can be discussed from organizational, societal and individual safety management perspectives (45). Multi-level engagement strategies are required to maximize athlete adherence to the programs. Hence, future studies should focus on enhancing performance programs to catch athletes' engagement in prevention strategies.

There is also a lack of studies regarding the epidemiology of football-related injuries, especially in women's football, and at both professional and amateur levels. Specific evidence is necessary to investigate at these levels, such as intrinsic (e.g., hormonal regulation, motivation for prevention programs' implementation) and extrinsic factors (e.g., climate, playing position, periods of fixed match congestion, number of matches and breaks per season), with the multiple stakeholders involved in football, so that the effectiveness of interventions can be tested.

The evidence regarding the use of preventive programs and measures are still scarce, therefore no definite conclusions for football stakeholders (e.g., coaches, medical or science departments) can be made. However, low levels of perceived risk may increase injury risk (22). Therefore, studies are needed to design and implement behavioural interventions to educate players, coaches, and other stakeholders about injury risk, aiming to explain the risks and consequences of injuries, and elucidate long-term costs for health and socio-economic systems.

### Limitations

There were small sample sizes across studies, the geographical areas of the studies were limited, the participants included in the studies were generally young, and studies with both sex (17, 18, 22, 25) did not include representative samples of both sexes, thus affecting the generalization of the findings.

Some of the questionnaires were not available in the athlete's native language and did not include open questions about their opinions. Also, in some studies, the questionnaires were completed in a team setting and might have been subject to social desirability bias in the team ambience.

Official medical records did not back up most studies about injury risk, and retrospective study designs may have increased the bias regarding the assessment of accurate injury history.

Moreover, a limitation of the current review is the possibility of a high risk of bias, as assessed in the JBI critical appraisal checklists, with studies lacking essential items of the studies’ methodology, and therefore results should be interpreted with caution.

## Conclusion

In conclusion, this systematic review explored football players' perceptions of injury risk and prevention. Most football players agreed that their risk of injury is high and that prevention strategies are important, still, they do not intend to use some of these strategies. It is crucial to acknowledge perceived injury risk factors, namely low muscle strength, lack of physical fitness, fatigue, excessive training and type and condition of surfaces, as well as injury prevention factors, such as the warm-up, workload monitoring and strength and conditioning training.

A better understanding of how the coach and medical departments match with players' perceptions may help inform delivery strategies, leading to better compliance with injury prevention programs. In addition, there is a need to alter the perceptions through education, rule changes, economic measures, and changes in the governance of the sport. Questioning more stakeholders and policymakers can shed light on such potential interventions.

## Data Availability

The original contributions presented in the study are included in the article/**Supplementary Material**, further inquiries can be directed to the corresponding author/s.
