# Supplementary material for: The perception of injury risk and prevention among football players: A systematic review
Source: Front Sports Act Living. 2022 Dec 7;4:1018752. doi: 10.3389/fspor.2022.1018752 (PMC9768495; doi:10.3389/fspor.2022.1018752)
Supplement: Supplementary file 2 [file Table2.docx]

**The perception of injury risk and prevention among football players: a systematic review**

**Supplementary file 2 - Full search strategy**

| **Database** | **Search** | **Limits** | **Results** |
| --- | --- | --- | --- |
| **PubMed** | ((("injury risk"[Title/Abstract] OR "injury prevention")[Title/Abstract]) AND ((perception[Title/Abstract] OR beliefs[Title/Abstract] OR knowledge[Title/Abstract] OR attitude)[Title/Abstract])) AND ((football[Title/Abstract] OR soccer)[Title/Abstract]) | Title/ Abstract | 168 |
| **Scopus** | ( TITLE-ABS-KEY ( ( "injury risk"  OR  "injury prevention" ) )  AND  TITLE-ABS-KEY ( ( perception  OR  beliefs  OR  knowledge  OR  attitude ) )  AND  TITLE-ABS-KEY ( ( football  OR  soccer ) ) ) | Title/ Abstract/ Keyword | 250 |
| **Web of Science** | (“injury risk” OR “injury prevention”) AND (perception OR beliefs OR knowledge OR attitude) AND (football OR soccer) | topic | 303 |
| **APA PsychINFO** | (“injury risk” OR “injury prevention”) AND (perception OR beliefs OR knowledge OR attitude) AND (football OR soccer) | Texto todo | 79 |
