# Supplementary material for: The perception of injury risk and prevention among football players: A systematic review
Source: Front Sports Act Living. 2022 Dec 7;4:1018752. doi: 10.3389/fspor.2022.1018752 (PMC9768495; doi:10.3389/fspor.2022.1018752)
Supplement: Supplementary file 3 [file Table3.docx]

**The perception of injury risk and prevention among football players: a systematic review**

**Supplementary file 3 – Risk of Bias in Studies**

| **JBI critical appraisal checklist** | | | | | | |
| --- | --- | --- | --- | --- | --- | --- |
|  | **Question** | **Yes** | **No** | **Unclear** | **NA** | **Overall appraisal** |
| **RCT** | | | | | | |
| Lorentsen et al, 2021 | 1 |  |  | X |  | Include |
|  | 2 |  |  | X |  |  |
|  | 3 |  |  | X |  |  |
|  | 4 |  | X |  |  |  |
|  | 5 |  | X |  |  |  |
|  | 6 |  |  |  | X |  |
|  | 7 |  |  |  | X |  |
|  | 8 |  | X |  |  |  |
|  | 9 | X |  |  |  |  |
|  | 10 | X |  |  |  |  |
|  | 11 | X |  |  |  |  |
|  | 12 |  | X |  |  |  |
|  | 13 |  |  | X |  |  |
| McKay et al.,  2014 | 1 | X |  |  |  | Include |
|  | 2 |  | X |  |  |  |
|  | 3 |  | X |  |  |  |
|  | 4 |  | X |  |  |  |
|  | 5 | X |  |  |  |  |
|  | 6 |  | X |  |  |  |
|  | 7 | X |  |  |  |  |
|  | 8 | X |  |  |  |  |
|  | 9 | X |  |  |  |  |
|  | 10 | X |  |  |  |  |
|  | 11 | X |  |  |  |  |
|  | 12 | X |  |  |  |  |
|  | 13 |  |  | X |  |  |
| **Cohort** | | | | | | |
| Loose et al.,  2018 | 1 |  |  |  | X | Include |
|  | 2 |  |  | X |  |  |
|  | 3 | X |  |  |  |  |
|  | 4 |  | X |  |  |  |
|  | 5 |  | X |  |  |  |
|  | 6 | X |  |  |  |  |
|  | 7 | X |  |  |  |  |
|  | 8 |  |  | X |  |  |
|  | 9 |  | X |  |  |  |
|  | 10 |  | X |  |  |  |
|  | 11 | X |  |  |  |  |
| Kontos et al.,  2004 | 1 |  |  |  | X | Include |
|  | 2 |  |  | X |  |  |
|  | 3 | X |  |  |  |  |
|  | 4 |  | X |  |  |  |
|  | 5 |  | X |  |  |  |
|  | 6 | X |  |  |  |  |
|  | 7 | X |  |  |  |  |
|  | 8 |  |  | X |  |  |
|  | 9 |  | X |  |  |  |
|  | 10 |  | X |  |  |  |
|  | 11 | X |  |  |  |  |
| **Cross-sectional** | | | | | | |
| Geertsema et al., 2021 | 1 | X |  |  |  | Include |
|  | 2 | X |  |  |  |  |
|  | 3 | X |  |  |  |  |
|  | 4 | X |  |  |  |  |
|  | 5 |  | X |  |  |  |
|  | 6 |  | X |  |  |  |
|  | 7 | X |  |  |  |  |
|  | 8 | X |  |  |  |  |
| Hawkins et al., 1998 | 1 |  | X |  |  | Include |
|  | 2 | X |  |  |  |  |
|  | 3 |  |  | X |  |  |
|  | 4 |  | X |  |  |  |
|  | 5 |  | X |  |  |  |
|  | 6 |  | X |  |  |  |
|  | 7 |  | X |  |  |  |
|  | 8 | X |  |  |  |  |
| Liporaci et al.,  2021 | 1 | X |  |  |  | Include |
|  | 2 | X |  |  |  |  |
|  | 3 |  |  | X |  |  |
|  | 4 | X |  |  |  |  |
|  | 5 |  | X |  |  |  |
|  | 6 |  | X |  |  |  |
|  | 7 | X |  |  |  |  |
|  | 8 |  | X |  |  |  |
| Zech et al.,  2017 | 1 | X |  |  |  | Include |
|  | 2 | X |  |  |  |  |
|  | 3 | X |  |  |  |  |
|  | 4 | X |  |  |  |  |
|  | 5 | X |  |  |  |  |
|  | 6 |  | X |  |  |  |
|  | 7 | X |  |  |  |  |
|  | 8 | X |  |  |  |  |
| McKay et al.,  2016 | 1 | X |  |  |  | Include |
|  | 2 | X |  |  |  |  |
|  | 3 | X |  |  |  |  |
|  | 4 | X |  |  |  |  |
|  | 5 |  | X |  |  |  |
|  | 6 |  | X |  |  |  |
|  | 7 | X |  |  |  |  |
|  | 8 | X |  |  |  |  |
| Alahmad et al., 2021 | 1 | X |  |  |  | Include |
|  | 2 | X |  |  |  |  |
|  | 3 | X |  |  |  |  |
|  | 4 | X |  |  |  |  |
|  | 5 | X |  |  |  |  |
|  | 6 |  | X |  |  |  |
|  | 7 | X |  |  |  |  |
|  | 8 | X |  |  |  |  |
| Som et al.,  2022 | 1 | X |  |  |  | Include |
|  | 2 | X |  |  |  |  |
|  | 3 | X |  |  |  |  |
|  | 4 | X |  |  |  |  |
|  | 5 |  | X |  |  |  |
|  | 6 |  | X |  |  |  |
|  | 7 |  | X |  |  |  |
|  | 8 | X |  |  |  |  |
| Weldon et al.,  2022 | 1 | X |  |  |  | Include |
|  | 2 | X |  |  |  |  |
|  | 3 | X |  |  |  |  |
|  | 4 | X |  |  |  |  |
|  | 5 |  | X |  |  |  |
|  | 6 |  | X |  |  |  |
|  | 7 | X |  |  |  |  |
|  | 8 |  | X |  |  |  |
| **Qualitative and mixed methods** | | | | | | |
| Mears et al.,  2018 | 1 | X |  |  |  | Include |
|  | 2 | X |  |  |  |  |
|  | 3 | X |  |  |  |  |
|  | 4 | X |  |  |  |  |
|  | 5 | X |  |  |  |  |
|  | 6 |  | X |  |  |  |
|  | 7 |  | X |  |  |  |
|  | 8 | X |  |  |  |  |
|  | 9 | X |  |  |  |  |
|  | 10 | X |  |  |  |  |
| O’Brien et al.,  2021 | 1 | X |  |  |  | Include |
|  | 2 | X |  |  |  |  |
|  | 3 | X |  |  |  |  |
|  | 4 | X |  |  |  |  |
|  | 5 | X |  |  |  |  |
|  | 6 |  | X |  |  |  |
|  | 7 | X |  |  |  |  |
|  | 8 | X |  |  |  |  |
|  | 9 | X |  |  |  |  |
|  | 10 | X |  |  |  |  |

JBI, Joanna Briggs Institute; NA, not applicable; RCT, randomized controlled trial.
